# Label-free functional analysis of root-associated microbes with dynamic quantitative oblique back-illumination microscopy

**DOI:** 10.1038/s41598-024-56443-1

**Published:** 2024-03-09

**Authors:** Caroline E. Serafini, Madison Green, Abigail Diering, Marcus T. Cicerone, Lily S. Cheung, Joel E. Kostka, Francisco E. Robles

**Affiliations:** 1 George W. Woodruff School of Mechanical Engineering, Georgia Institute of Technology, Atlanta, GA 30318 USA; 2 School of Biological Sciences, Georgia Institute of Technology, Atlanta, GA 30318 USA; 3 School of Chemistry and Biochemistry, Georgia Institute of Technology, Atlanta, GA 30332 USA; 4 School of Chemical and Biomolecular Engineering, Georgia Institute of Technology, Atlanta, GA 30332 USA; 5 Wallace H. Coulter Department of Biomedical Engineering, Georgia Institute of Technology and Emory University, Atlanta, GA 30318 USA

**Keywords:** Label-free imaging, Nitrogen fixation, Quantitative phase imaging, Optics and photonics, Optical techniques, Imaging and sensing, Microbial ecology, Fluorescence imaging, Optical imaging, Optics and photonics, Optical techniques, Imaging and sensing, Microbial ecology, Fluorescence imaging, Optical imaging

## Abstract

The increasing global demand for food, coupled with concerns about the environmental impact of synthetic fertilizers, underscores the urgency of developing sustainable agricultural practices. Nitrogen-fixing bacteria, known as diazotrophs, offer a potential solution by converting atmospheric nitrogen into bioavailable forms, reducing the reliance on synthetic fertilizers. However, a deeper understanding of their interactions with plants and other microbes is needed. In this study, we introduce a recently developed label-free 3D quantitative phase imaging technology called dynamic quantitative oblique back-illumination microscopy (DqOBM) to assess the functional dynamic activity of diazotrophs in vitro and in situ. Our experiments involved three different diazotrophs (*Sinorhizobium meliloti*, *Azotobacter vinelandii*, and *Rahnella aquatilis*) cultured on media with amendments of carbon and nitrogen sources. Over 5 days, we observed increased dynamics in nutrient-amended media. These results suggest that the observed bacterial dynamics correlate with their metabolic activity. Furthermore, we applied qOBM to visualize microbial dynamics within the root cap and elongation zone of *Arabidopsis thaliana* primary roots. This allowed us to identify distinct areas of microbial infiltration in plant roots without the need for fluorescent markers. Our findings demonstrate that DqOBM can effectively characterize microbial dynamics and provide insights into plant-microbe interactions *in situ*, offering a valuable tool for advancing our understanding of sustainable agriculture.

## Introduction

An expanding world population and the need for economic growth are increasing the demand for food, fuel, and renewable feedstocks. Nitrogen availability often limits crop yields^1^–a problem typically counteracted through the application of synthetic fertilizers. But despite their widespread use, almost half of the nitrogen added through chemical fertilizers is lost to the surrounding environment, resulting in cascades of adverse ecological and societal effects^2^. Consequently, new approaches are needed to improve nitrogen use efficiency, balance the global nitrogen cycle, and make agriculture more sustainable.

Nitrogen-fixing bacteria, known as diazotrophs, naturally reside in the rhizosphere and offer a process that can be leveraged to reduce the need for synthetic fertilizers. Diazotrophs associate with plants symbiotically and commensalistically by converting atmospheric nitrogen, N$_2$, into biologically usable forms such as NH$_3$. In addition to providing fixed nitrogen, diazotrophs improve plant productivity and biomass by influencing phytohormone levels in plants^3,4^. In return, plants provide fixed carbon compounds like sugars and organic acids to the bacteria. In addition to the exchange of metabolites and signaling molecules, plants support diazotrophs in other ways, most notably by creating root nodules that provide a favorable microaerobic environment for nitrogen fixation in legumes. Whether equivalent root morphological features favor nitrogen fixation by non-symbiotic endophytic diazotrophs in other plant species remains an open question^5,6^.

In order to leverage microbial nitrogen fixation to develop more sustainable agricultural practices, more research is needed to understand the complex interactions between plants and their soil partners^7,8^. The study of these interactions has currently relies on molecular and genetic analyses^9–12^. Although genomics and proteomics provide an understanding of gene and protein expression in this system, they employ destructive techniques that can not yet provide real-time or holistic spatial information on microbial dynamics in situ. As such, imaging modalities have emerged as a tool to study root colonization. These imaging tools primarily consist of brightfield microscopy^13–15^ and fluorescence-based imaging^9,12,13,16^. While brightfield microscopy is easy to implement, it alone lacks the contrast needed to visualize structural details of plant and bacterial cells. Consequently, the microbial cells are often tagged with fluorescent markers or stained histochemically for imaging. However, these methods suffer from a number of limitations, including photobleaching, phototoxicity, and biological tissue alterations from the exogenous agents. Thus, there remains a need for a label-free imaging modality capable of characterizing microbial inoculation of plant samples in situ.

**Figure 1:**
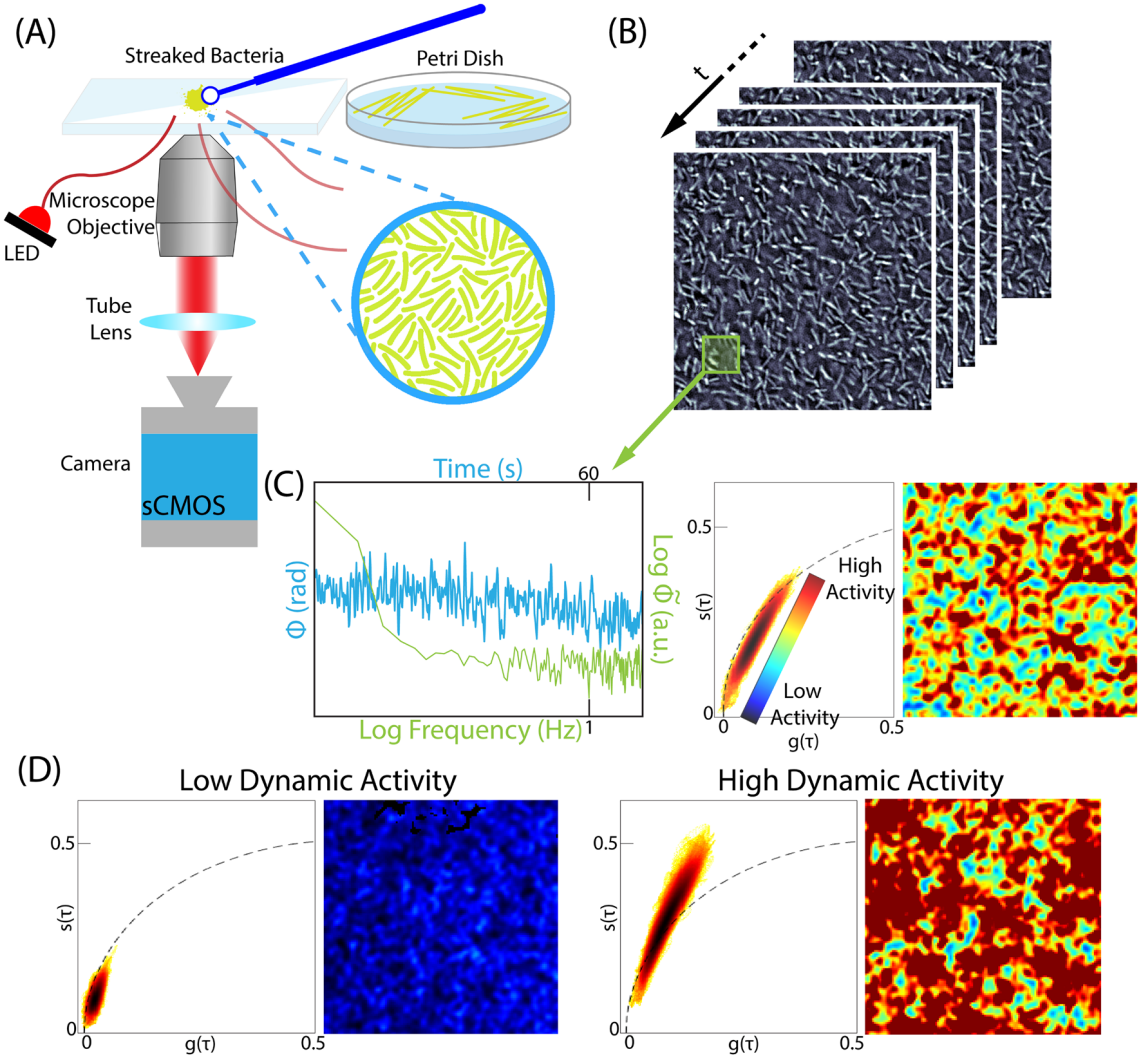
(**A**) Overview of the qOBM imaging approach which consists of an inverted brightfield microscope with epi-illumination. The sample (cultured bacteria streaked onto a glass slide) is sequentially illuminated by 4 optical fibers connected to 720 nm LEDs, followed by quantitative phase recovery. (**B**) Time-lapsed qOBM image stack of cultured *Azotobacter vinelandii* bacteria. (**C**) Blue: temporal phase fluctuations of a pixel corresponding to the boxed area of bacteria in (**B**). Green: log-log representation of the Fourier transform of the temporal phase value. This plot is used to generate the phasor plot (middle) and the colorized DqOBM image (right) demonstrating areas of high and low dynamics. (**D**) Representative phasor plots and DqOBM images of areas with low dynamics (left) and high dynamics (right).

Quantitative Oblique Back-illumination Microscopy (qOBM) is a novel, label-free, low-cost, non-invasive optical imaging modality capable of providing information on the functional dynamic activity and metabolic state of cultured cells, in vivo and in situ^17–19^. qOBM enables 3D quantitative phase imaging (QPI) with epi-illumination. Like QPI^20–24^, qOBM provides clear, quantitative contrast of cellular and subcellular structures based on refractive index properties (which are linearly proportional to dry mass), but with the significant and unique advantage that qOBM can do so in 3D, in thick scattering samples with same-side epi-illumination (Fig. 1A)^17–19,25^. While this methodology has been utilized to observe eukaryotic cells (i.e. blood cells^26^, cell cultures^18,25^, and neuronal tissues^17,27,28^), this paper provides a novel use of functional qOBM imaging to study bacterial cells.

To enable functional imaging with qOBM, we have developed dynamic-qOBM (DqOBM)^25^, which ultimately yields a functional map depicting temporal changes in the refractive index (and thus dry mass) that correlate to patterns of cellular and sub-cellular dynamics within the sample. Specifically, in DqOBM, a sample is imaged over a period of time, and the resulting temporal signal for each spatial pixel is analyzed based on its temporal frequency response, which exhibits an exponential-like behavior that can be characterized using phasor analysis, as illustrated in Fig. 1 (more details in the “Results” and “Methods” section). Here, we analyze microbial processes in the range of 0.1 Hz up to 4 Hz, thus our analysis may include information from dynamics such as flagella-driven motility^29,30^, cell growth, division, proliferation, nitrogen fixation and respiration. Further, because $<0.01$ Hz dynamics are omitted, the DqOBM signal is insensitive to slower processes such as osmotic transport, plant exudates, energy-storage granule formation, and plant cell division. The final DqOBM image obtained through this analysis is a functional map, depicting temporal changes in the refractive index (and thus dry mass) that correlate to patterns of cellular and sub-cellular dynamics within the sample (Fig. 1C,D).

Here, we show the ability of DqOBM to assess the dynamics of known nitrogen-fixing bacteria both in vitro and in situ in *Arabidopsis thaliana* roots. First, we show that DqOBM is capable of measuring changing dynamics in bacteria cultured with various amounts of nitrogen and carbon over several days. Second, we use DqOBM to identify spatial patterns of bacterial colonization in *Arabidopsis thaliana* primary roots. We also compare the DqOBM dynamics with multi-mode quantitative phase-fluorescence microscopy used to detect microbe inoculation in roots. These results demonstrate the potential of DqOBM to serve as a label-free and non-destructive imaging modality capable of detecting microbial activity and endophytic behavior in plant roots. This work paves the way for qOBM, and/or other imaging tools that can assess refractive index/dry-mass dynamic in scattering samples, to provide a deeper understanding of nitrogen-fixing diazotrophs in natural soil environments.

## Results

### DqOBM analysis of bacteria culture dynamics in vitro

**Figure 2:**
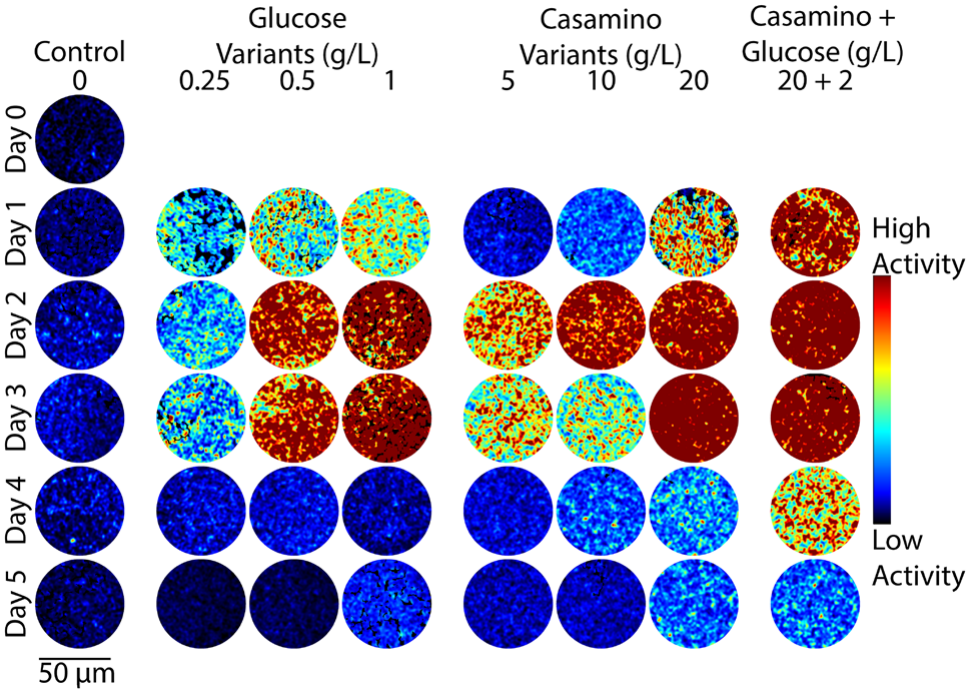
Representative colorized DqOBM images of the nitrogen-fixing bacteria *A. vinelandii*. Columns represent culture treatments with varying carbon and nitrogen concentrations. Rows indicate the time expired relative to inoculation.

**Figure 3:**
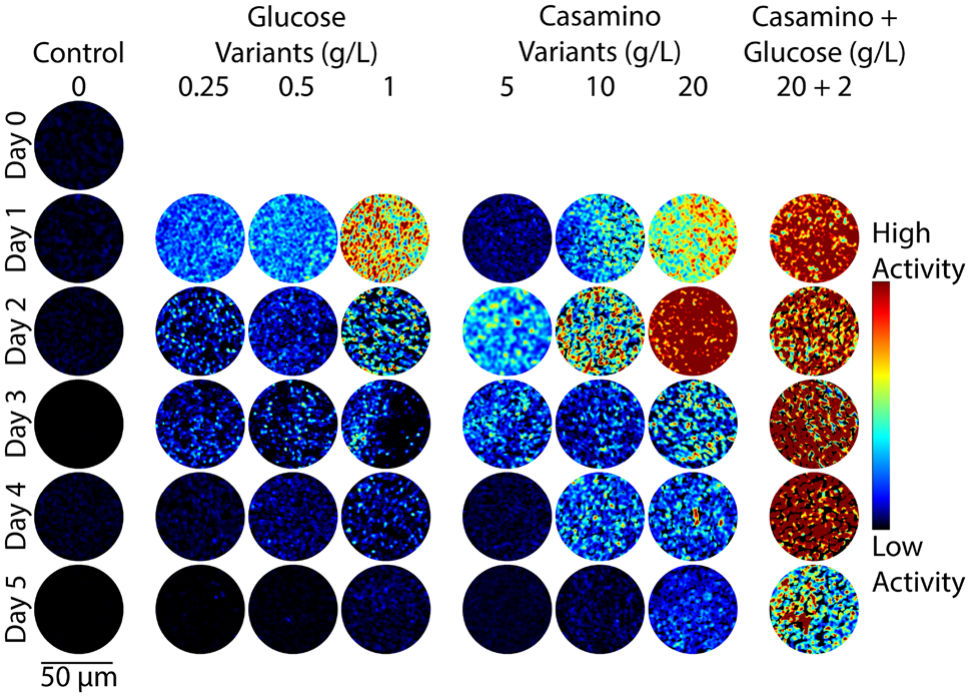
Representative colorized DqOBM images of the nitrogen-fixing bacteria *Rahnella aquatilis*. Columns represent culture treatments with varying carbon and nitrogen concentrations. Rows indicate the time expired relative to inoculation.

**Figure 4:**
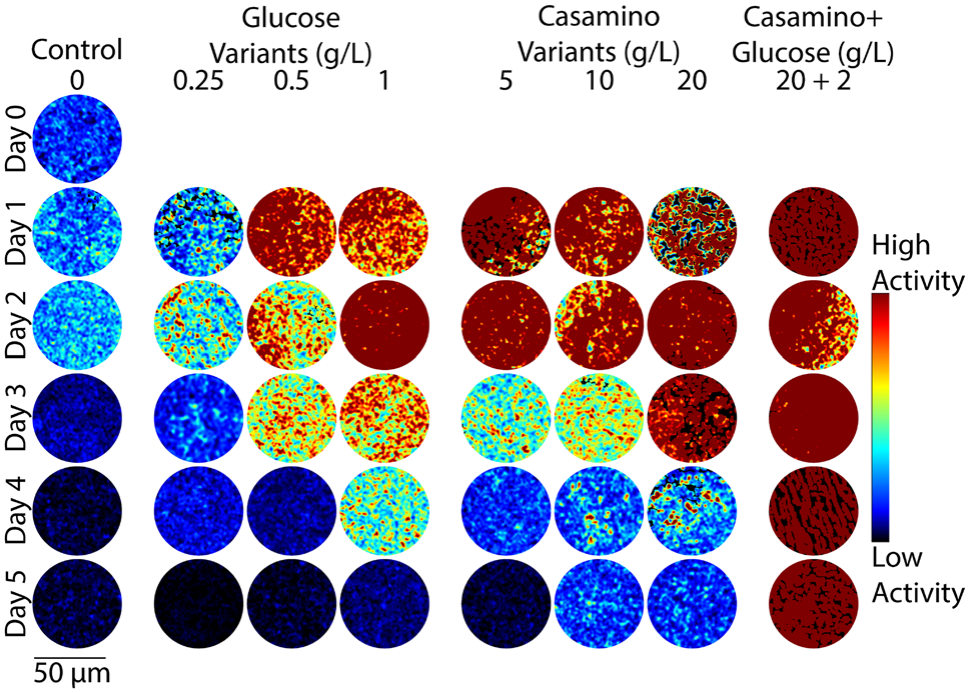
Representative colorized DqOBM images of the nitrogen-fixing bacteria *Sinorhizobium meliloti*. Columns represent culture treatments with varying carbon and nitrogen concentrations. Rows indicate the time expired relative to inoculation.

To test the hypothesis that the dynamics measured with DqOBM reflects microbial activity, we broadly perturbed cell state by modulating nutrient availability in cultures. The plated media treatments included additions of glucose (0.25 g/L, 0.5 g/L, 1 g/L), casamino acids (5 g/L, 10 g/L, 20 g/L), or a combination of both (20 g/L casamino acids, 2 g/L glucose). Carbon substrate was already present in our base media; thus, small concentrations of glucose were used. Casamino acids served as a source of organic nitrogen compounds, and it was previously shown that the chosen concentrations of 5–20 g/L of casamino acids lead to the repression of nitrogenase activity^31,32^. DqOBM images were collected for different diazotrophs (*Azotobacter vinelandii*, *Rahnella aquatilis*, and *Sinorhizobium meliloti*) grown in the eight media treatments over 5 days. Observed dynamics of *A. vinelandii* increased with additions of both glucose and casamino acids, with peak dynamics detected 2–3 days after plating (Fig. 2). *S. meliloti* and *R. aquatilis* also exhibit increased microbial dynamics with increases in carbon and nitrogen-containing compounds (Figs. 3 and 4), but the highest dynamics were generally observed 1–2 earlier.

The observed results are in line with expectations. All bacteria cultured with high concentrations of glucose should have experienced a general increase in energy availability and thus increased microbial activity^33^, resulting in higher dynamic responses (Figs. 2, 3, and 4). Similarly, nitrogen fixation is metabolically expensive^34^, thus we expect to see an increase in microbial dynamics when the competing energy demand for nitrogenase activity is reduced due to a surplus of fixed nitrogen from casamino acids. Results in Figs. 2, 3, and 4 show that, indeed, when bacteria are no longer required to expend energy fixing atmospheric nitrogen, rates of other microbial processes increase. These processes may include flagella-driven motility^29,30^, cell growth, division, and respiration—all of which are encapsulated within the DqOBM signal. Increases in microbial dynamics due to increases in available energy are further supported by the high dynamics recorded when excess carbon and nitrogen-containing compounds are supplied together and by the lack of microbial dynamics recorded when excess compounds were not provided (Figs. 2, 3, and 4).

Temporal patterns were observed as an initial rise in dynamics followed by a slow decrease over time, mirroring growth patterns (Figs. 2, 3, and 4). *R. aquatilis* grows faster than *A. vinelandii* or *S. meliloti* and thus consumes its nutrients faster, which is reflected in the large reduction of dynamics on day 3 for *R. aquatilis* compared with day 4 or 5 for *A. vinelandii* and *S. meliloti*, respectively (Figs. 2, 3, and 4). All phasor plots and end-member decays from the phasor plots of the different cultured plates can be seen in Supplemental Section S1. It is important to note that we currently lack the ability to distinguish which exact set of microbial dynamic processes are being measured with DqOBM. Again, we simply define microbial dynamics as any combination of the following: metabolism, growth, proliferation, nitrogen fixation, motility, and any other dynamic cellular processes. Additionally, it is unclear whether the temporal patterns we captured are caused by the changes in the same processes for the different bacteria.

Figures 2, 3, and 4 provide a spatial representation of the dynamics, with red signifying higher dynamics and blue lower. A quantitative analysis of these DqOBM images can be accomplished by computing the total signal energy (Eq. 1) captured in the frequency response of the dynamic signals. This can be accounted for by taking the sum of the area under the curve of the phasor line decay signal (as seen in the green line in Fig. 1C)^35^. We then integrate the phase signal from 0.1 Hz (to remove the DC component of the signal) to 4 Hz (half of our sampling frequency). This is:

1 $$\begin{aligned} E_{signal}=\sum _{0.1\,\text {Hz}}^{4\,\text {Hz}}\left| \widetilde{\phi }(f) \right| ^{2} \end{aligned}$$

**Figure 5:**
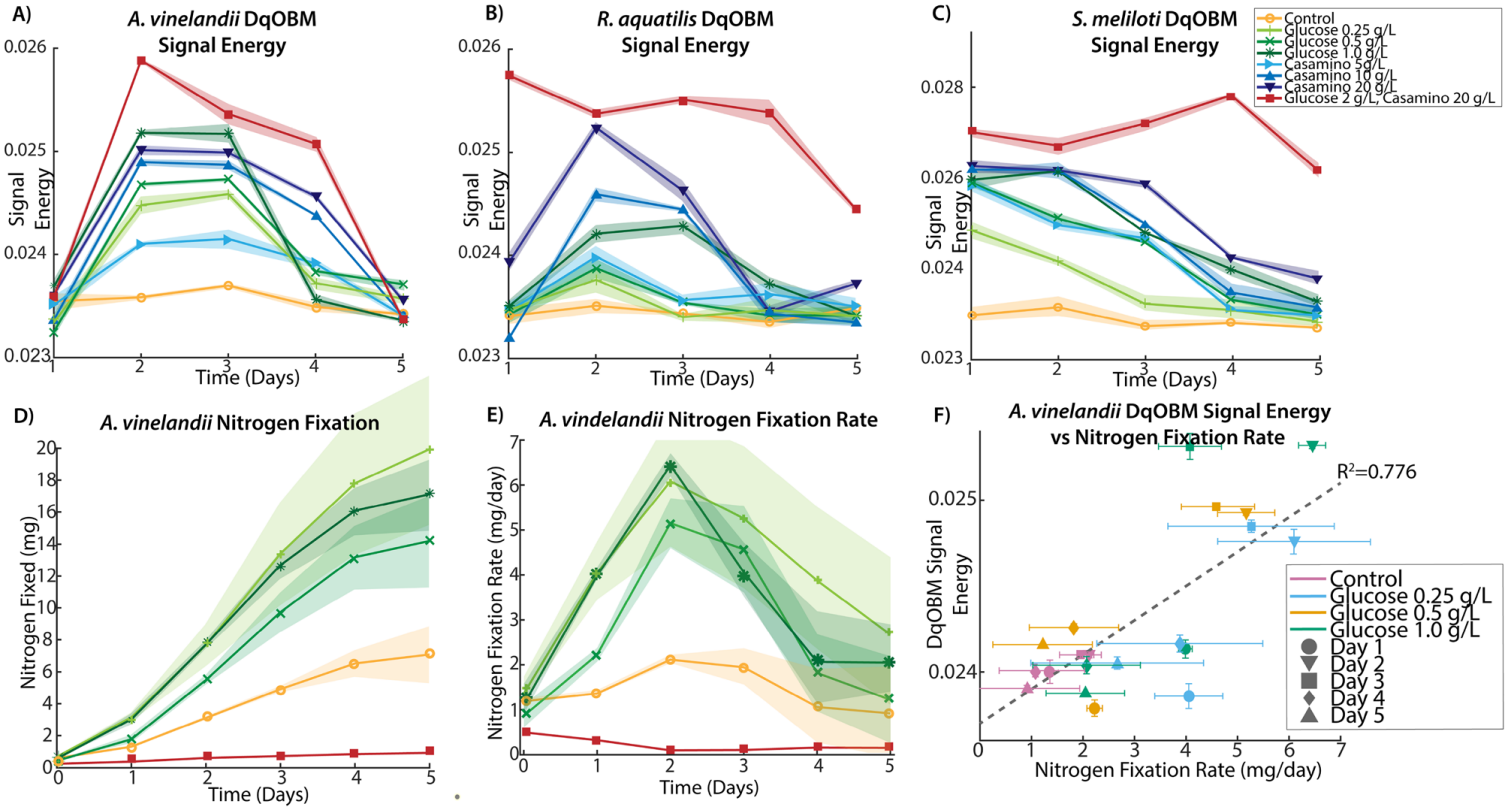
The signal energy over time of *A. vinelandii*, *R. aquatilis* and *S. meliloti*, respectively. (**A**) Shows a sharp decline to a dormant state of the glucose variants between D3–4 whereas the casamino variants show continued signal energy. (**B**) Shows some increased dynamics from the casamino acid groups. (**C**) Shows increased prolonged dynamics for the casamino acid *S. meliloti* variants. Standard deviation in the plot is demonstrated from the shaded area surrounding the lines and represents the standard deviation of the signal energy from three replicates. (**D**) Shows the nitrogen fixation values from the ARA study using *A. vinelandii* microbes. (**E**) Shows the derivative of the plot in (**D**) to show the nitrogen fixation rate. (**F**) Shows a comparison of the DqOBM energy with the nitrogen fixation rate.

The summation of the signal energy via Eq. (1) quantifies the decay signals visualized in Figs. 2, 3, and 4 and Supplemental Section S1. Plots of the signal energy can be seen in Fig. 5A–C.

For this signal energy calculation, we take the frequency response of the most dynamic signal within a group, as determined by the endmember of the phasor plot (Supplemental Section S1). These results allow us to graphically (instead of pictorially) visualize the dynamics of a culture over time. The highest signal energy was observed in cultures amended with glucose and casamino acids (Fig. 5A–C). Further, the lowest dynamics observed was from cultures grown in Jensen’s media without any nutrient additions. To further establish the significance of the quantitative signal energy analysis, we conducted an ANOVA test revealing high significance (P<0.0001) for the effect of the culture age, plated nutrients, and the interaction for all cultures. A full report of the ANOVA values can be found in Supplemental Section 5, Table S1.

In Fig. 5A, the peak signal energy of *A. vinelandii* was observed after 2 days in cultures supplemented with glucose. This peak was followed by a sharp decline in signal energy between Days 3–4 of culture. In contrast, *A. vinelandii* produced a higher and more prolonged period of dynamics when cultured in media containing casamino acids additions. These observations are consistent with the growth rate and nitrogen fixation data found in the Supplemental Section S2. As discussed above, we hypothesize that differences in signal energy may be the result of enhanced energy conservation through respiration stimulated by supplements of carbon and nitrogen-containing substrates. The initial increase in signal energy reflects the use of the added substrates which provide the bacteria with greater amounts of energy since abundant nutrients are available and nitrogen fixation is not required. The decline following peak signal energy implies that the added carbon and nitrogen compounds have been depleted and the ability of cells to produce/conserve energy is diminished. Similarly for *R. aquatilis*, in Fig. 5B, high dynamics are observed for cultures with glucose and casamino acids additions followed by a return to dormancy. Here, we do not see the same increased glucose dynamics. This may be due to the fast growth of *R. aquatilis* which means that the sampling of *R. aquatilis* at D2 is more similar to D4 of the *A. vinelandii* (this fast growth rate is also seen in the growth rate curves in Supplemental Section S2). In future experiments, imaging at earlier time points may reveal similar peaks as seen in Fig. 5A. Finally, in Fig. 5C with *S. meliloti*, a similar pattern is observed as in Fig. 5A with *A. vinelandii*—high dynamics are observed media with high glucose and high casamino acid additions. Differences observed between growth rates of *A. vinelandii*, *R. aquatilis*, and *S. meliloti* under the same conditions, are likely due to a variety of factors including nutrient utilization, cell size and composition, and cell morphology. Future experiments comparing growth rate and DqOBM imaging with mass-balanced carbon and nitrogen amendments may shed light on the mechanisms underpinning differences in growth.

To better understand the processes captured with DqOBM, we performed nitrogen fixation studies on *A. vinelandii* (seen in Fig. 5D,E). Here, we look at the ethylene concentration produced by the microbes as a proxy for measuring nitrogen fixation rates. When analyzing the nitrogen fixation rate (Fig. 5E), we see a curve with a remarkably similar shape to the signal energy captured with DqOBM. To quantify this, we plotted the nitrogen fixation rate by the DqOBM signal energy. We observe a linear correlation with an R$^2$ value of 0.776, intimating a high level of correlation between the rates of microbial nitrogen fixation and signal energy. It is important to note that this signal is only observed for microbes grown media without fixed nitrogen from casamino acids. In the presence of nitrogen amendments, microbes cease carrying out nitrogen fixation. While these microbes still exhibit a high signal energy, we can attribute this signal energy to other microbial activities including motility and proliferation. While we are not yet able to directly link DqOBM dynamic responses to nitrogen fixation, we do show that dynamics observed with DqOBM imaging are nitrogen and carbon-limited and contain a strong linear correlation with the nitrogen-fixation rate of *A. vinelandii*.

### DqOBM imaging of bacteria in inoculated *Arabidopsis thaliana* roots

**Figure 6:**
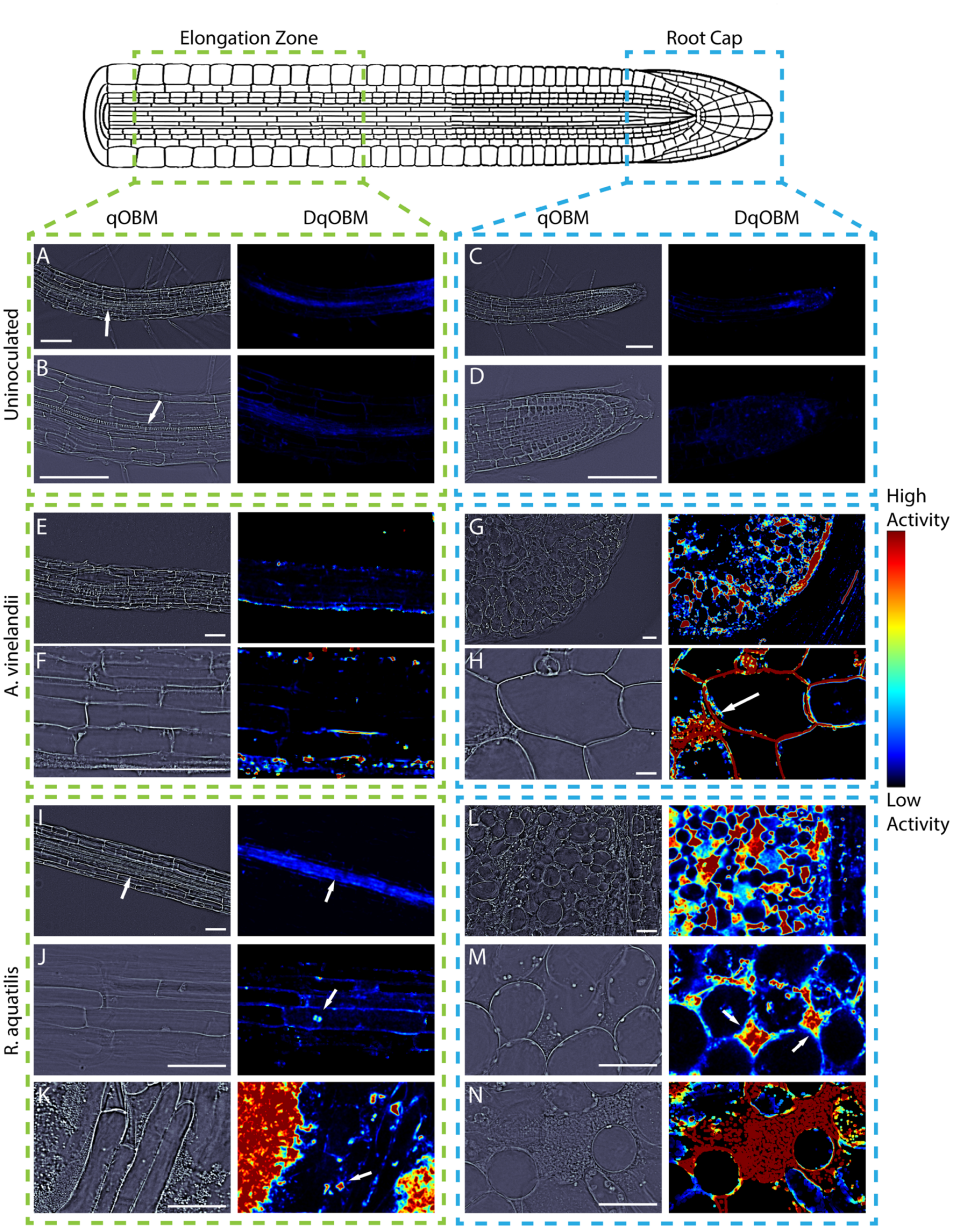
Representative qOBM (left) and DqOBM (right) images from uninoculated *A. thaliana* plants (**A**–**D**), *A. thaliana* inoculated with *A. vinelandii* (**E**–**H**) and *A. thaliana* inoculated with *R. aquatilis* (**I**–**N**). We look at regions from the elongation zone (**A**,**B**, **E**,**F**, and **I**–**K**) and from the root cap (**C**,**D**, **G**,**H**, and **L**–**N**). In the uninoculated plant (**A**–**D**), we see minimal fluctuations in the phase of the images, corresponding to low levels of dynamics. Similarly, (**E**) and (**F**) show the elongation zone inoculated by *A. vinelandii* with minimal dynamics. (**I**) demonstrates increased dynamics (as indicated by the arrow) in the center of the root, which may be indicative of nutrient flow through the xylem rather than microbial dynamics. (**J**) and (**K**) show isolated dynamic areas from individual microbial cells in the elongation zone and higher dynamics and microbe inoculation in the root cap (indicated by arrows in **J** and **K**). Further, (**K**) shows high dynamics from a large number of bacteria growing outside of the root. In the root cap, we see colonization of intercellular spaces as indicated by the arrows in (**H**) and (**M**) and the red in (**N**). All samples were prepared from uncut roots. All scale bars are 100 $\upmu$m.

After establishing that DqOBM provides quantitative information on microbial activity, our next goal was to image colonized roots. The *Arabidopsis thaliana* primary root, a well-studied organism, acted as our model experimental system. Seedlings were inoculated with either *A. vinelandii* or *R. aquatilis* and imaged 7 days post-inoculation. DqOBM images were taken along primary roots at the elongation zone and root cap, as illustrated in Fig. 6. As a control, uninoculated plants were also imaged (Fig. 6A–D).

Our results show that the uninoculated plants have low levels of dynamics. Using our qOBM modality, we were able to capture midsagittal optical sections of the primary root and visually identify the endodermal cells encasing the xylem and phloem that constitute the vasculature, as indicated by arrows in Fig. 6A,B,I. Next, using the DqOBM modality, we could show that the dynamics are higher in the root vasculature than in the surrounding cell layers (Fig. 6A,B), likely due to the transport of water, minerals, and nutrients. This vasculature flow can further be seen in Supplementary Video V1, which contains a side-by-side visualization of the DqOBM image (Fig. 6A) with its corresponding time-lapse video of the phase images (scale bar: $100\,\upmu$m, frame rate: 30 fps). Here, we see low levels of dynamics.

In the elongation zone, many of the same patterns were observed in the inoculated and uninoculated plants—low overall levels of dynamics within the endodermal region, as seen in Fig. 6E,F,I. Further, in the plants inoculated with *R. aquatilis*, we observed small areas of high dynamics from microbial cells that have colonized into the elongation zone. These are indicated by the white arrows in Fig. 6J,K. In K, microbial cells located outside of the plant root exhibit high dynamics. This can be seen in Supplemental Video V2 containing an endodermal region of the root (corresponding to Fig. 6K) surrounded by high levels of microbial activity just outside of the root (scale bar: $100\,\upmu$m, frame rate: 30 fps). These results are indicative of qOBM’s ability to detect the microbial dynamics and the sparsity of microbial cells that have infiltrated more centrally into the root. In other areas of the elongation zone, microbial cells are either not present in the qOBM image or show lower dynamics and thus are not seen in the DqOBM image.

For comparison, we also imaged the root cap, which has been shown to be the first point of contact for bacterial cells attempting to colonize the root^36^. Additionally, the root cap may act as a filter that prevents further microbial colonization in the root^37^. Here, we would expect to see increased microbial dynamics due to increased infiltration of bacteria. Indeed, in both the plant inoculated with *A. vinelandii* and *R. aquatilis*, we observe higher dynamics than in the uninoculated plant or in the elongation zone, as seen in Fig. 6G,H,L–N. Dynamics, and by association microbial cells, are predominantly present in intercellular spaces (white arrows in Fig. 6M and the red in N). In Supplemental Videos V3 and V4, we can also see high dynamics corresponding to the DqOBM images seen Fig. 6L,M (scale bar: $100\,\upmu$m, frame rate: 30 fps). These show time-lapse videos of the dynamics exhibited in the intercellular spaces. This level of increased dynamics suggests higher levels of plant-secreted carbon compounds in the root cap attracting bacteria and encouraging replication. Such a possibility is consistent with reports of higher production of arabinogalactan-proteins and certain organic acids at root tips^38,39^, which can serve as carbon and energy sources for the bacteria. Phasor plots from the colonized roots can be seen in Supplemental Section S3.

These results are well-aligned with previous studies of root colonization. For example, *R. aquatilis* has been shown to colonize concavities and root hairs in corn^40^. *Gluconacetobacter diazotrophicus*, another free-living diazotroph, has been shown to penetrate and colonize the intracellular space in *A. thaliana*. The colonization begins at root meristems and emergence points of lateral roots. Colonization by *G. diazotrophicus* has also been observed in *A. thaliana* xylem^41^. *Bacillus subtilis* has demonstrated similar behavior in *A. thaliana*^42^. The intracellular colonization of these bacteria suggests that the dynamics seen in the DqOBM images can indeed be the result of the intracellular colonization by *A. vinelandii* and *R. aquatilis*. Further, the morphology in Supplemental Videos V3 and V4 represents the dynamics of the colonizing bacteria.

**Figure 7:**
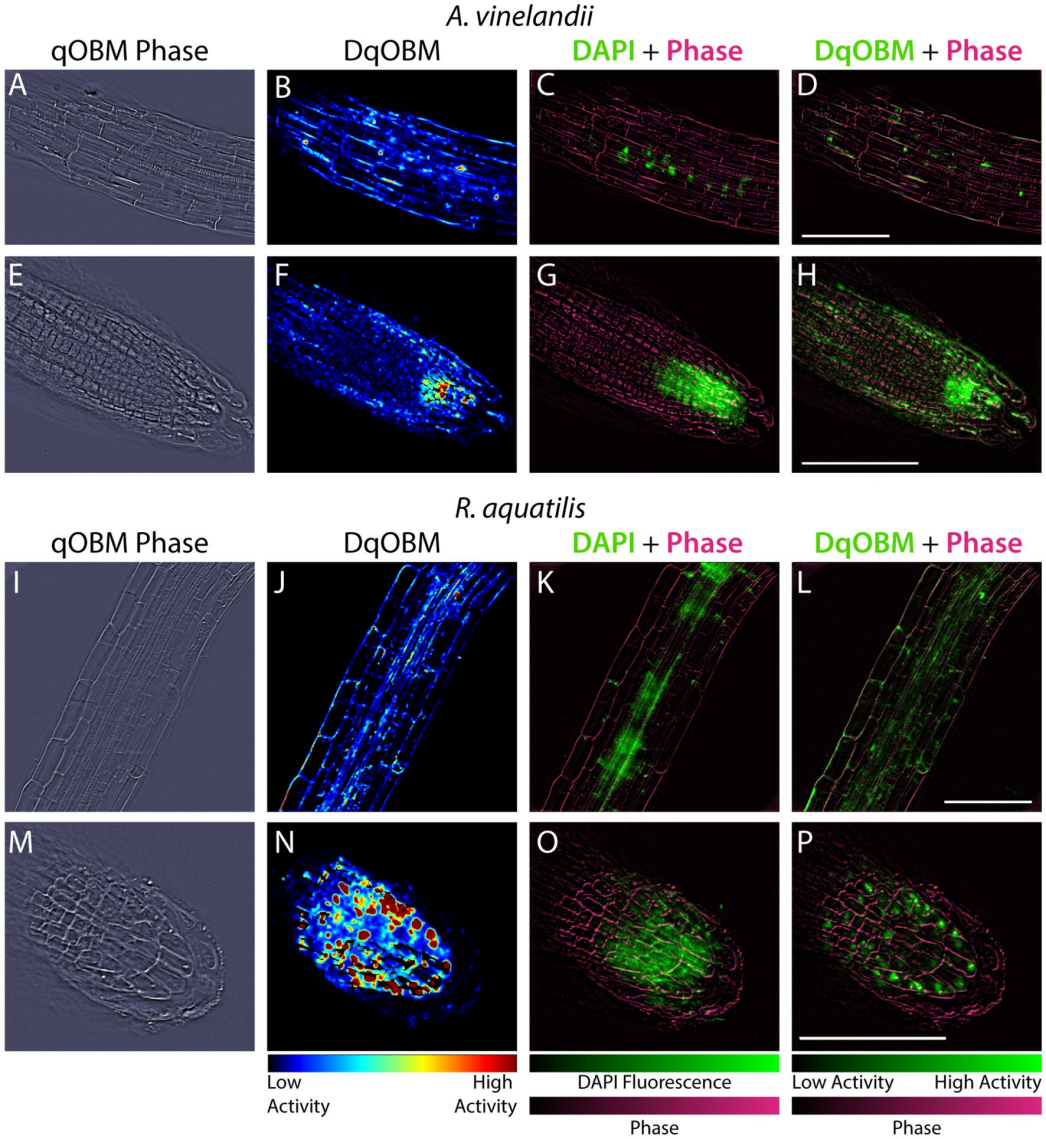
DAPI-labeled bacteria in plants. (**A**–**H**) Show *A. vinelandii* inoculated plants. (**I**–**P**) Show *R. aquatilis* inoculated plants. (**A**,**E**,**I**,**M**) Show qOBM phase images, (**B**,**F**,**J**,**N**) Show DqOBM images, (**C**,**G**,**K**,**O**) Show DAPI fluorescence images (green) overlaid on qOBM phase images (magenta), and (**D**,**H**,**L**,**P**) Show DqOBM images (green) overlaid on qOBM phase images (magenta). All scale bars are 100 $\upmu$m.

Finally, to confirm that regions of high dynamics measured with DqOBM in plants indeed correspond to the bacteria, we converted the qOBM microscope into a multimodal system with an added fluorescence channel to excite 4$'$,6-diamidino-2-phenylindole (DAPI). As seen in Figs. 6 and 7, qOBM and DqOBM imaging can be used to extract structural phase information based on the refractive index distribution (Fig. 7A,E,I,M) and functional dynamic information (Fig. 7B,F,J,N). To confirm that regions of measured high dynamics with DqOBM in plants indeed correspond to the bacteria, we labeled the bacteria with DAPI. These fluorescence images (green channel) can be overlaid on the phase (or refractive index) qOBM images (magenta channel) as seen in Fig. 7C,G,K,O. To compare the DAPI fluorescence images with DqOBM, a similar overlay method was employed in Fig. 7D,H,L,P, where the DqOBM can be visualized in green and the phase images in magenta. Again, we notice that less *A. vinelandii* is observed in the elongation zone (Fig. 7B–D) than in the root cap (Fig. 7F–H) in both the DqOBM and DAPI images. Similar results could be observed with *R. aquatilis*, albeit the overall levels of dynamics, and consequently bacteria, were higher in these samples. We note that the images obtained in D, H, L, and P closely resemble those seen with current imaging modalities like confocal imaging^9^; however, unlike confocal, qOBM is a label-free analysis. We also see some minor differences between the bright field fluorescence and DqOBM images (K &L and O &P respectively) where the DAPI-stained image shows higher, more diffuse fluorescence than in the DqOBM images. We believe this points to the differences in axial sectioning of the images. In DqOBM, the axial resolution is on the order of 2–3 $\upmu$m (depending on the microscope objective used). With the DAPI bright field fluorescence imaging, there are no cross-sectional capabilities; therefore, the fluorescence would be summed from across the entire depth of light penetrating into the sample. Thus, these images may not present a perfect one-to-one match, but they show clear similarities to confirm that DqOBM regions of high dynamics correspond to the active bacteria. Phasor plots from the roots colonized with the labeled bacteria can be seen in Supplemental Section S4.

## Discussion

This paper presents DqOBM as a label-free imaging modality capable of monitoring microbial activity in real-time. No prior label-free imaging modalities have been able to provide the level of detail seen with qOBM in situ. Using DqOBM imaging, we were able to observe microbial dynamics in vitro and in situ during and following plant colonization. Additionally, we were able to observe changes in microbial dynamics under various carbon and nitrogen substrate amendments. This preliminary work paves the way for future experiments which may elucidate the underlying mechanism underlying carbon and nitrogen exchange.

Our resulting qOBM and DqOBM approach provides an opportunity to characterize free-living bacterial behavior in a system comparable to their natural environment. We again note that the signal energy level measured by DqOBM is likely a sum of all microbial activities. As such, research is needed to directly link dynamics observed by imaging with rates of growth or nitrogen fixation. Nonetheless, the trends in dynamics observed here are consistent with a dependence of energy level on carbon and nitrogen, and we demonstrate strong linear correlations between the nitrogen fixation and the DqOBM signal energy.

In situ experiments using *A. thaliana* allow us to observe complex plant-microbe interactions non-invasively. While the approach is unable to differentiate between the different types of microbial activities or the different bacteria types, DqOBM imaging allows for the visualization of “hotspots,” which we believe colocalize with sites of active polysaccharides and organic acids secretion. The sustainable optimization of bioenergy crop yields requires a better understanding of nutrient transport and metabolic transport in the rhizosphere, and the method described here represents a novel tool with which to gain new insights into these complex processes. In future experiments, we may be able to distinguish different DqOBM microbial dynamics by studying genetically mutated bacteria or using inhibitors to isolate particular microbial metabolic signals. These potential experiments include the use of non-motile mutants to study the effect of bacterial motility, nif mutants that have lost the ability to fix nitrogen to study the effect of metabolic nitrogen fixation, and specific antibiotics to inhibit microbial proliferation to study the signal of cell division.

In this paper, we utilized an imaging rate of 0.1–4 Hz. We opt to begin the analysis with 0.1 Hz to remove the DC component of the signal. Further, we can analyze up to 4 Hz (half our sampling frequency). While the top end of our frequency analysis is ultimately limited by the speed of our camera, we can use this to distinguish the dynamics detected. The window we analyze contains relatively fast, microscale dynamics that would correspond to bacterial motility, cell signaling, and protein expression. By limiting our analysis to this window, we can also exclude slower plant processes such as osmotic transport, plant exudation, energy-storage granule formation, and plant cell division^43^. Further, while we focus our analysis here on a 0.1–4 Hz sampling speed, future experiments may opt for faster or slower imaging speeds to capture different dynamic processes.

Lastly, while DqOBM provides the label-free functional imaging of bacterial cells in roots, the penetration depth of qOBM has been shown to be limited to $\sim$ 200 $\upmu$m in tissues^28^. Therefore, it will not be able to penetrate deep into the natural environment of roots, such as soil. Nevertheless, this work demonstrates that dynamics, as measured by temporal fluctuations of phase (or refractive index), can be applied as a surrogate to visualize bacteria and their dynamics in plants. We expect that new imaging modalities like qOBM and DqOBM will eventually provide a better understanding of metabolic exchange between plants and their microbiome and catalyze advances in crop biotechnology and agricultural practices for a sustainable future

## Methods

### Bacteria growth conditions and techniques

Three bacterial cultures were used in this study: *Rahnella aquatilis* strain OV588 was kindly gifted to us by our collaborators at Oak Ridge National Research Laboratory, the *Sinorhizobium meliloti* strain SM1021 was generously provided by Sharon R. Long, and the *Azotobacter vinelandii* which was isolated from peatlands in northern Minnesota/obtained from ATCC (Catalog number BAA-1303). To study nitrogen fixation activity, the bacteria were continuously cultured on Jensen’s nitrogen-free media. Cultures were kept on Jensen’s plates in a room-temperature incubator between experiments.

Carbon and nitrogen-containing compounds were varied through the addition of glucose and casamino acids to the media. To prepare media containing different concentrations of glucose and casamino acids, fresh nitrogen-free Jensen’s with agar was prepared using Milli-Q water. Three glucose concentrations (0.25, 0.5, and 1 g/L) were added to freshly prepared media before being autoclaved. Similarly, three casamino acid concentrations (5, 10, and 20 g/L) were added to fresh media. In addition, unaltered Jensen’s media was used as a control. To observe maximum dynamics, media containing large amounts of glucose (2 g/L) and casamino acids (20 g/L) was prepared. Cultures of *S. meliloti, A. vinelandii,* and *R. aquatilis* were carried out in triplicate started. Microbial dynamics were then measured using qOBM imaging every 24 h for 5 days. Sterile inoculation loops were used to select colonies from each plate and to lightly streak colonies onto glass slides. Streaking was implemented to create a uniform layer of cells and colonies of like size were selected for imaging. Once the colonies were streaked out, a cover slip was placed on the glass slide.

### Plant growth conditions and techniques

*Arabidopsis thaliana* wild-type (ecotype Columbia) seed was generously provided by A K M Mahmudul Huque and Lily Cheung. Seeds were surface sterilized with 2% sodium hydroxide along with 0.05% Triton X-100 for 5 min and then washed five times with sterile water. Seeds were then kept at 4 °C for 2 days for vernalization. Seeds were grown in liquid 1/2 strength nitrogen-free Murashige and Skoog media supplemented with 1% (w/v) sucrose in a 6-well plate at 23 °C and 50% relative humidity under 16 hr-light/8 hr-dark conditions for 7 days. All in-planta experiments were conducted with 7-day-old seedlings. The plant collection and use was in accordance with all the relevant guidelines.

### Bacterial growth and inoculation

To prepare for inoculation, *S. meliloti*, *A. vinelandii*, and *R. aquatilis* cultures were grown to an OD of 1 to 1.5 at 28 °C in liquid Jensen’s nitrogen-free media. Before inoculation, each culture was pelleted through centrifugation and washed in Milli Q water before being resuspended in Milli Q water. The bacterial suspensions were diluted to 10% and then used to inoculate either *M. truncatula* or *A. thaliana* roots on nitrogen-free MS plates.

### DAPI staining

To perform DAPI staining, *A. vinelandii* and *R. aquatilis* cultures were grown up to an OD of 1.5 before being washed and resuspended in PBS. A 1 $\upmu$g mL$^{-1}$ DAPI solution was added to the inoculum and the mixture was incubated for 3 min in the dark. *A. thaliana* roots were rinsed with distilled water and airdried on membrane filters. Following incubation with DAPI, the 10 $\upmu$L of inoculum was then pipetted onto and used to innoculate *A. thaliana* roots.

### Acetylene reduction assay

*A. vinelandii* was grown in 5 mL of each media treatment (Jensen’s, 0.25 g/L glucose, 0.5 g/L glucose, 1 g/L glucose, 5 g/L casamino acids, 10 g/L casamino acids, 20 g/L casamino acids, and 2 g/L glucose 20 g/L casamino acids) for 3 days at 25 °C. Cells were washed, harvested and diluted to 10% before being added to fresh aliquots of the amended media treatments in 30 mL serum bottles. Serum bottles were sealed to be gas-tight and then flushed with argon for 10 min. The serum bottle headspace was injected with 10% vol oxygen and 10% vol acetylene. Nitrogen fixation activity was then measured using the acetylene reduction assay method based on acetylene reduction into ethylene by nitrogenase^44^. Ethylene concentrations were measured over a 5-day time series using gas chromatography.

### Growth curve culture

Bacteria were precultured in eight media treatments (Jensen’s, 0.25 g/L glucose, 0.5 g/L glucose, 1 g/L glucose, 5 g/L casamino acids, 10 g/L casamino acids, 20 g/L casamino acids, and 2 g/L glucose 20 g/L casamino acids). Fresh aliquots of amended media were then inoculated with precultured cells at a 10% dilution in triplicate. Cultures were then loaded onto a 96-well plate and the plate was loaded into a BioTek Synergy H1 microplate reader where cultures were kept at 25 °C and continually shaken. OD600 was measured at 30-min intervals over 5 days.

### qOBM imaging system

The qOBM setup comprises a conventional brightfield microscope integrated with a modified illumination arrangement, as outlined in previous studies^17–19,26^. In contrast to the traditional transmission-based illumination utilized in both brightfield microscopy and QPI, the qOBM approach employs an epi-mode illumination strategy using a quartet of LED light sources emitting at 720 nm. These LEDs are strategically positioned around the objective at $90^{\circ }$ intervals, as depicted in Fig. 1A, and are coupled via optical multimode fibers. In the epi-mode configuration, $\sim$ 45 mW of light is directed onto the sample. Within the sample, photons undergo multiple scattering interactions, resulting in changes in their trajectory, with a subset being redirected back toward the microscope objective. This phenomenon effectively establishes a virtual light source within the sample, leading to oblique back-illumination, a phenomenon previously termed oblique back-illumination^45^. Fluctuations in the refractive index across the sample guide the light either towards or away from the microscope objective. This refractive index-induced modulation induces intensity variations that encode the sample’s refractive index properties. In our investigations, we employ Nikon S Plan Fluor LWD 20X (numerical aperture 0.45) and Nikon S Plan Fluor LWD 40X (numerical aperture 0.6) objectives. The light collected by the microscope is captured by an sCMOS camera (pco.edge 4.2 LT).

To achieve quantitative phase imaging with qOBM, we first subtract the intensity images obtained from opposing illumination angles (these intermediate images are called differential phase contrast or DPC images). Subsequently, two orthogonal DPC images, acquired through a total of four distinct acquisitions, are deconvolved using the optical transfer function of the system. This process, previously elaborated upon in Refs.^17–19,26,27^, ultimately yields quantitative phase contrast images. Leveraging the rich quantitative phase information acquired, we achieve the capability to both visualize and quantify cellular and subcellular structures within the sample, enabling comprehensive tracking of their developmental progression over time.

### Imaging procedure

To assess whether or not we could use qOBM dynamics to assess microbial dynamics, we first imaged the bacteria plates over the course of 5 days (D0–5) with 8 different glucose and nitrogen concentrations. Each day, representative streaks were taken from each plate and restreaked onto a glass slide. On each slide, 2 different fields of view were taken with 600 images at 8 Hz. This was repeated for each bacteria type (*A. vinelandii*, *R. aquatilis*, and *S. meliloti*).

Next, we went to image plants inoculated with the same bacteria strains. *A. thaliana* was inoculated with *R. aquatilis* and *A. vinelandii* and imaged at D1 and D7 after inoculation. We studied FOV with inoculated and uninoculated roots and with bacteria outside of the root. Again the same dynamic imaging procedure was followed collecting 600 images at 8 Hz.

### Dynamic image analysis

To conduct functional imaging with qOBM, we have developed Dynamic qOBM (DqOBM)^25^. In DqOBM a sample is imaged over a period of time—for all samples in this work this time period is 75 s at 8 Hz to obtain 600 images. From these images, we obtain a pixel-wise dynamic frequency response given by the absolute value of the Fourier transform of the temporal phase signal, $\widetilde{\phi }(f) = | \mathscr {F} \{ \phi ( t ) \} |$ for each spatial pixel in the image. We observe that the frequency response of the pixels exhibits an exponential or multi-exponential decay indicative of subcellular mass movement that more prominently oscillates at low frequencies and dampens exponentially with increasing frequency. This functional behavior is expected for cell structures such as cell membranes^46^ and mitochondria^47^, among other structures^48^. As expected, the dynamic response in background regions shows a mostly flat near-zero amplitude dynamic response, indicative of static behavior.

To visualize the cell dynamics, we utilize phasor analysis^49,50^, as it aligns well with the exponential nature of the dynamic phase frequency response, $\widetilde{\phi }(f)$. Phasor analysis is a widely used technique for examining spectral and dynamic signals, especially those with exponential characteristics, such as those encountered in fluorescent lifetime and pump-probe microscopy^49,50^. This method involves decomposing signals into two variables, typically referred to as g and s, which are derived from the cosine and sine transforms (real and imaginary components of the Fourier Transform) of the dynamic signals (here $\widetilde{\phi }(f)$) for each spatial pixel in the image at a particular period, $\tau$. In our case, we choose $\tau$=0.5 s depending on the net acquisition rate to decompose the signals into g and s following Eqs. (2) and (3), respectively:

2 $$\begin{aligned} &  g_i\left( \tau \right) = \frac{\int \widetilde{\phi _i}\left( f \right) {\textrm{cos}}\left( 2\pi f \tau \right) {\textrm{d}}f}{\int \widetilde{\phi _i}\left( f \right) {\textrm{d}}f} \end{aligned}$$

3 $$\begin{aligned} &  s_i\left( \tau \right) = \frac{\int \widetilde{\phi _i}\left( f \right) {\textrm{sin}}\left( 2\pi f \tau \right) {\textrm{d}}f}{\int \widetilde{\phi _i}\left( f \right) {\textrm{d}}f} \end{aligned}$$

In phasor space, the two components, g and s, act as coordinates, uniquely defining each pixel in the image. As a result, the phasor plot represents a 2D histogram that captures the distribution of g and s values across the image. Similar dynamic signals tend to cluster together in this space, while mixtures of exponential signals create linear mappings between regions. The boundary points of these distributions are termed endmembers.

The cumulative phasor plot in Fig. 1C,D shows signals that lie mostly within the universal semicircle (black dotted line in Fig. 1C,D), which signifies that the frequency response of the cellular dynamics, for the most part, follows an exponential behavior^49,50^. But because the frequency response is not physically constrained to be strictly exponential, some signals are observed outside the universal semi-circle (but they are within the unit circle due to the normalization of g and s). Figure 1C,D also shows the average responses from two distinct regions in phasor space.

The dynamic signal energy was calculated in accordance with Eq. (1). This signal energy is represented at the area under the curve of a phasor line decay. In this paper, we integrate the signal from 0.1–4 Hz.

### Statistical analysis

To compare the quantitative dynamic signal energy analysis conducted in Fig. 5, we conducted a two-factor ANOVA analysis with replication. The ANOVA analysis conducted looked at the effect of the colony age, plate nutrients, and the interaction effect between age and plate nutrients. Fisher’s LSD tests were conducted with a P-value < 0.05 considered significant. All groups contained 3 samples.

Further, in Fig. 5F, we show a plot comparing the nitrogen fixation rate with the DqOBM energy. We perform a line fitting using a linear regression. We report an R$^2$ value to demonstrate the goodness of fit of that line.

## Supplementary Information

Supplementary Information.

Supplementary Video 1.

Supplementary Video 2.

Supplementary Video 3.

Supplementary Video 4.

## Data Availability

Data underlying the results presented in this paper are not publicly available at this time but may be obtained from the corresponding author upon reasonable request.
