# Supplementary material for: Label-free functional analysis of root-associated microbes with dynamic quantitative oblique back-illumination microscopy
Source: Sci Rep. 2024 Mar 9;14:5812. doi: 10.1038/s41598-024-56443-1 (PMC10925023; doi:10.1038/s41598-024-56443-1)
Supplement: Supplementary file 1 — Supplementary Information. [file 41598_2024_56443_MOESM1_ESM.pdf]

Supplemental Material

Supplemental Section 1: Phasor Plots of Cultured Bacteria and Endmember Decays

Below are the phasor plots and endmember decays for the cultured *Azotobacter vinelandii*, *Rahnella aquatilis*, and *Sinorhizobium meliloti* bacteria. These data points correspond to the DqOBM images in Figures 2, 3, and 4.

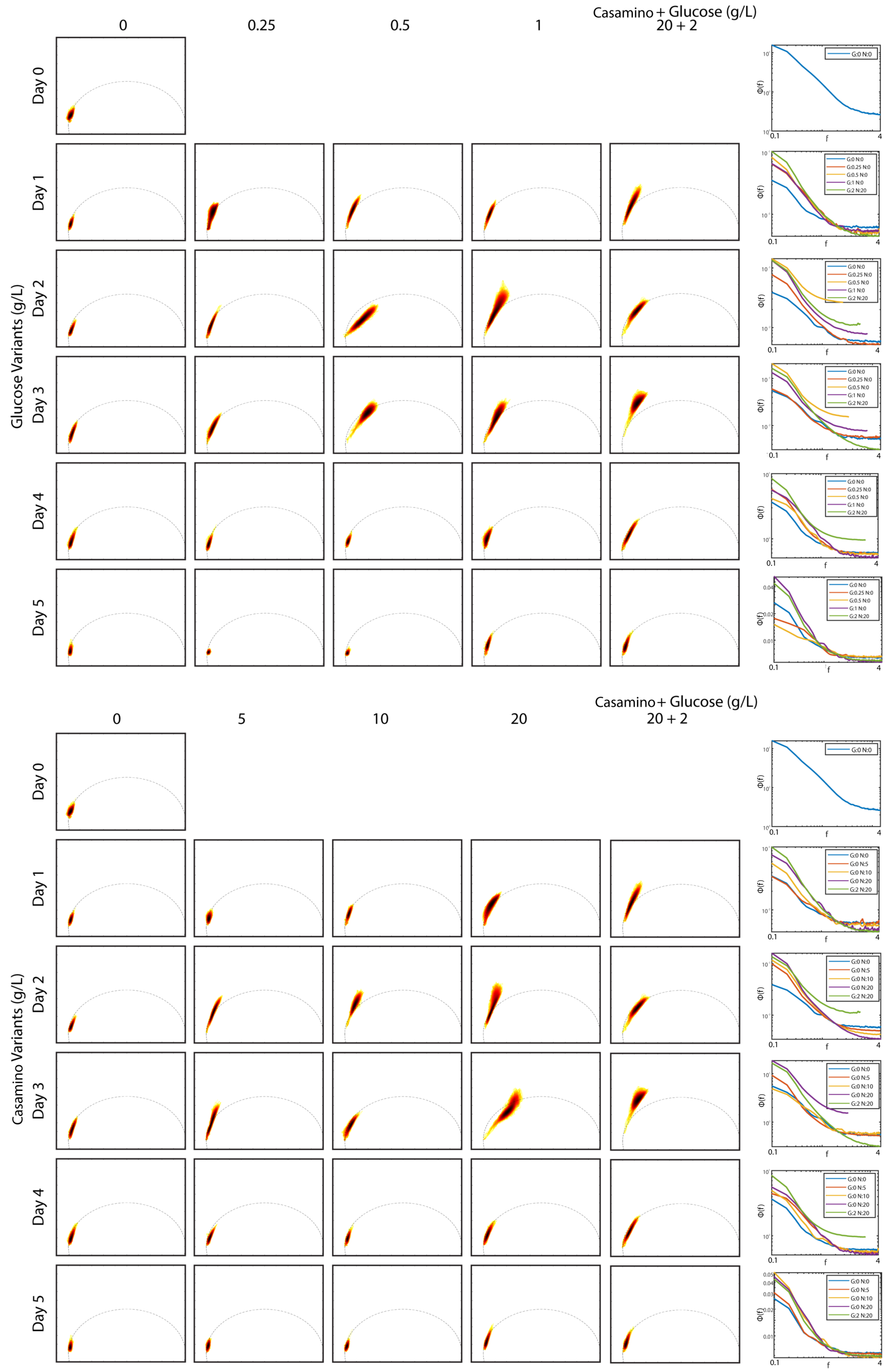

Figure S1. *Azotobacter vinelandii* Phasor Plots and Endmembers

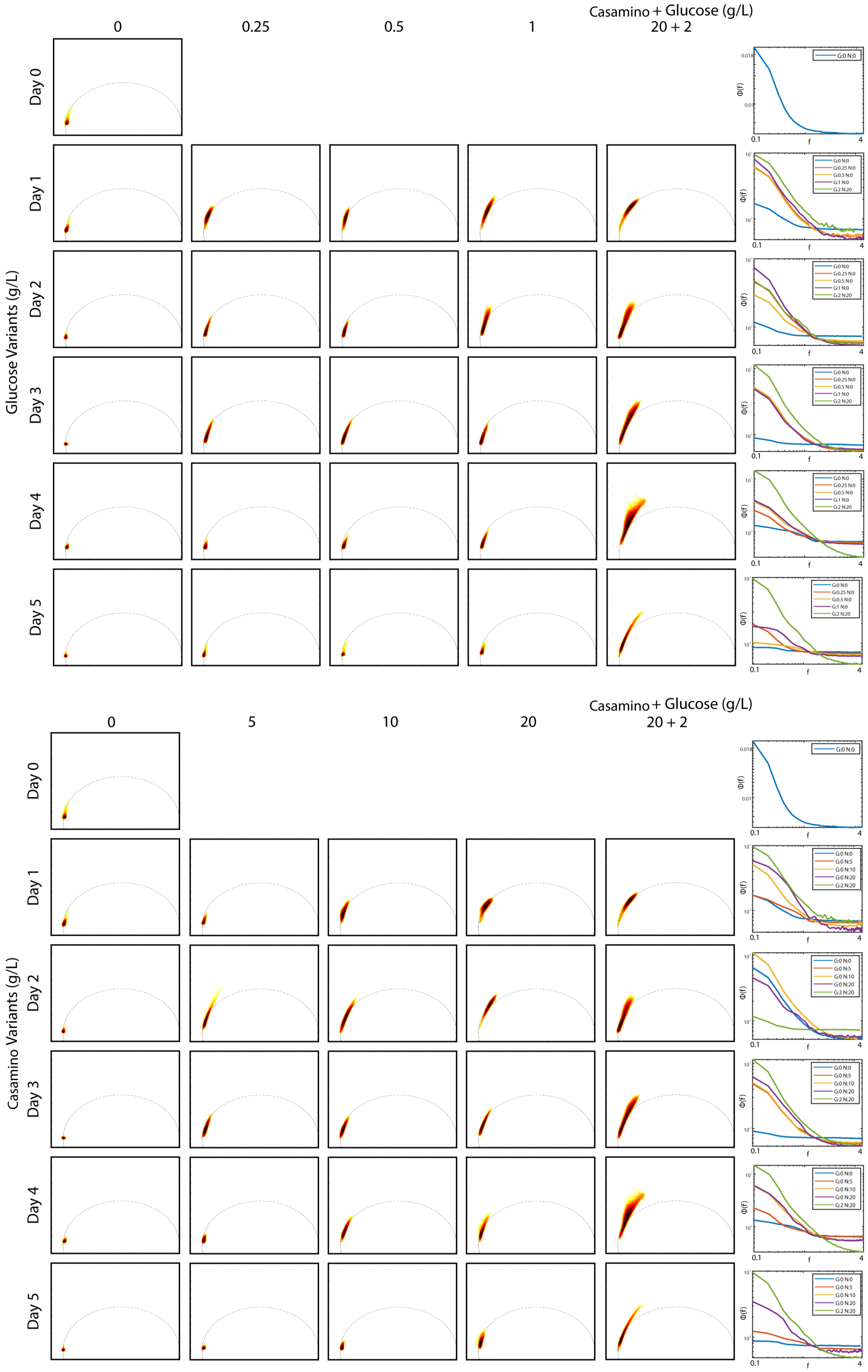

Figure S2. *Rahnella aquatilis* Phasor Plots and Endmembers

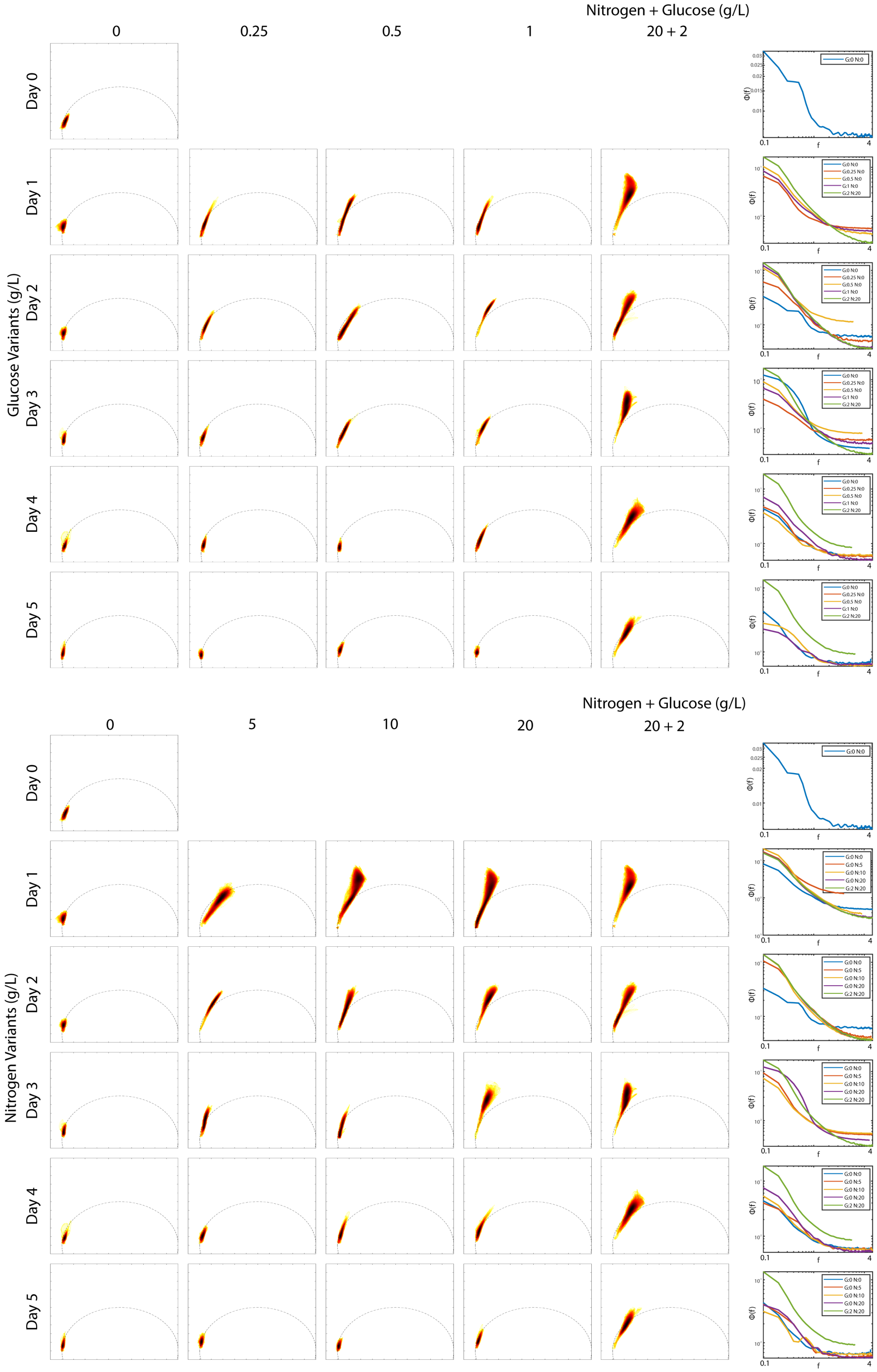

Figure S3. *Sinorhizobium meliloti* Phasor Plots and Endmembers

Supplemental Section 2: Growth Curves of Cultured Bacteria

As expected, optical density and growth rate values were highest for bacterial cultures given casamino acids (A & B). In comparison, additions of glucose led to less significant increases in growth rate. The addition of casamino acids resulted in a total repression of nitrogenase and little to no nitrogen fixation. Inversely, additions of glucose led to increased nitrogen fixation even compared to the control. As previously mentioned, this system is saturated with carbon which may explain the absence of stepwise increases in nitrogen fixation with increases in glucose concentrations. The resulting growth and nitrogen fixation rates for *A. vinelandii* show an inverted relationship with carbon and nitrogen additions. Increases in casamino acids concentration produce an increase in growth rate but a significant decrease in nitrogen fixation. We believe these results support our hypothesis that increases in dynamic activity are due to increases in energy conservation coming from additional carbon substrate and the repression of nitrogen fixation. Trends in dynamic activity are similar to trends observed in growth rate and nitrogen fixation rates, and we note that the DqOBM curves seen in Fig. 5 are the result of a sum of microbial activities.

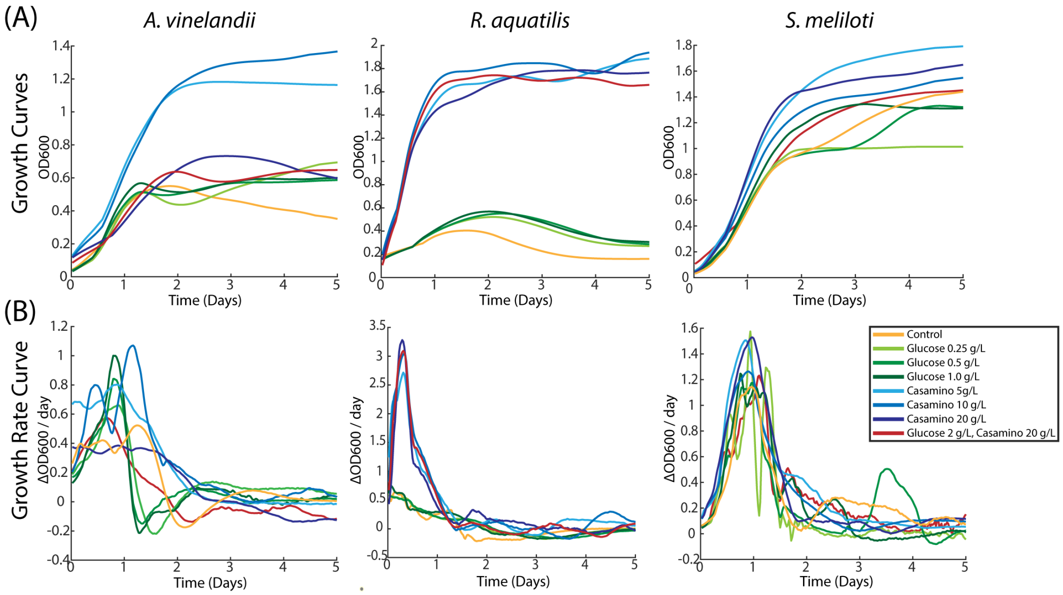

Figure S4. (A) Growth curves for *A. vinelandii*, *R. aquatilis*, and *S. meliloti* from D0-5. (B) Growth rate curves for *A. vinelandii*, *R. aquatilis*, and *S. meliloti* from D0-5.

Supplemental Section 3: Phasor Plots of Plant Structures

Below are the phasor plots for the images in Fig. 6.

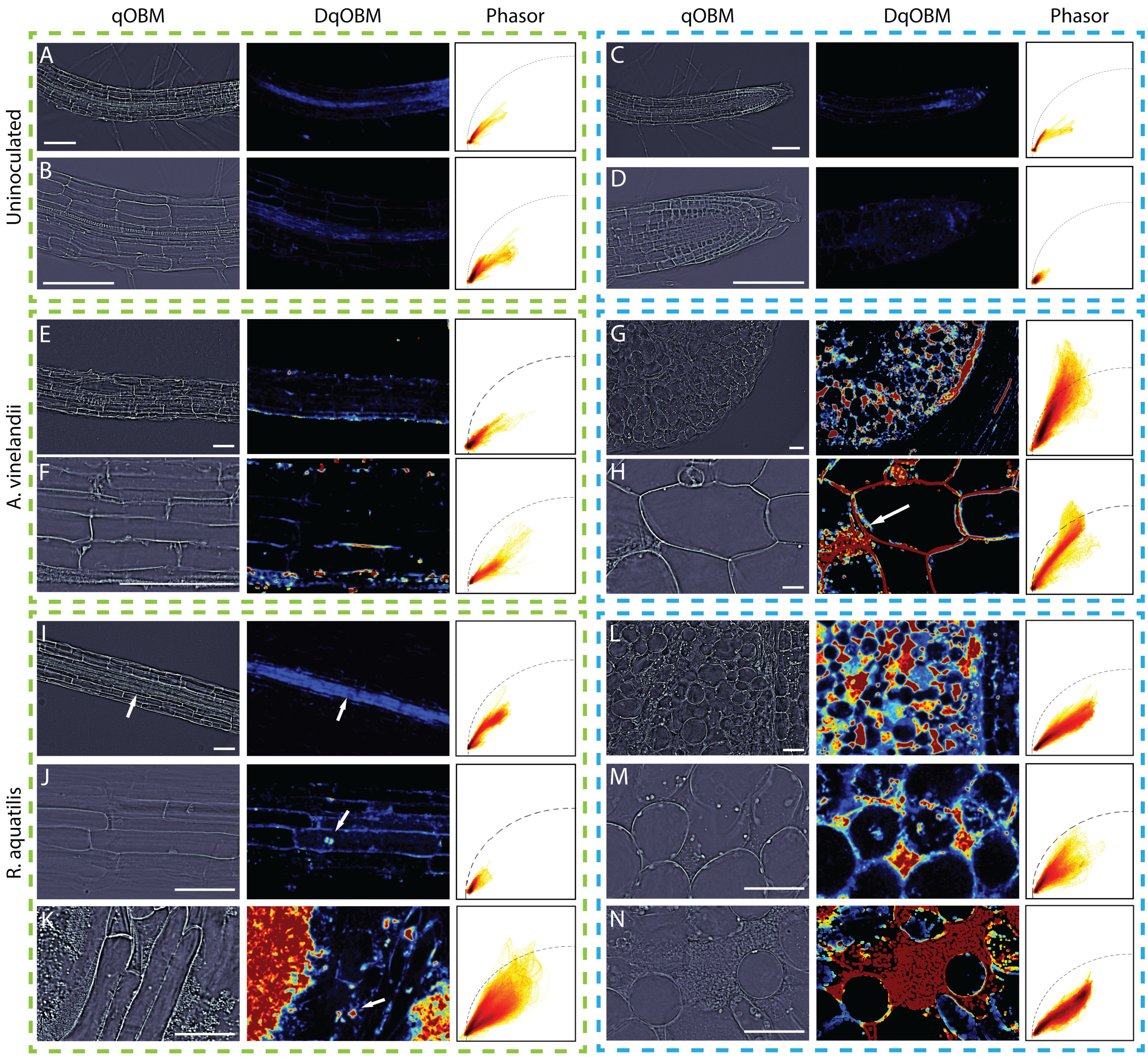

Figure S5. As described in the main text, we see little dynamic activity in the phasor plots of A-D of the uninoculated plant. We see increase activity in G & H in the rootcap of *A. vinelandii* inoculated Arabidopsis and little activity in the elongation zone. In I, we see dynamic activity likely from the xylem. In J &K, we see isolated microbial activity (indicated by arrows). K also shows high dynamic activity outside of the root. Lastly, L-N we see multi-dynamic activity with the intercellular bacteria exhibiting the higher dynamic activity.

Supplemental Section 4: Phasor Plots of Plant Structures

Below are the phasor plots for the images in Fig. 7.

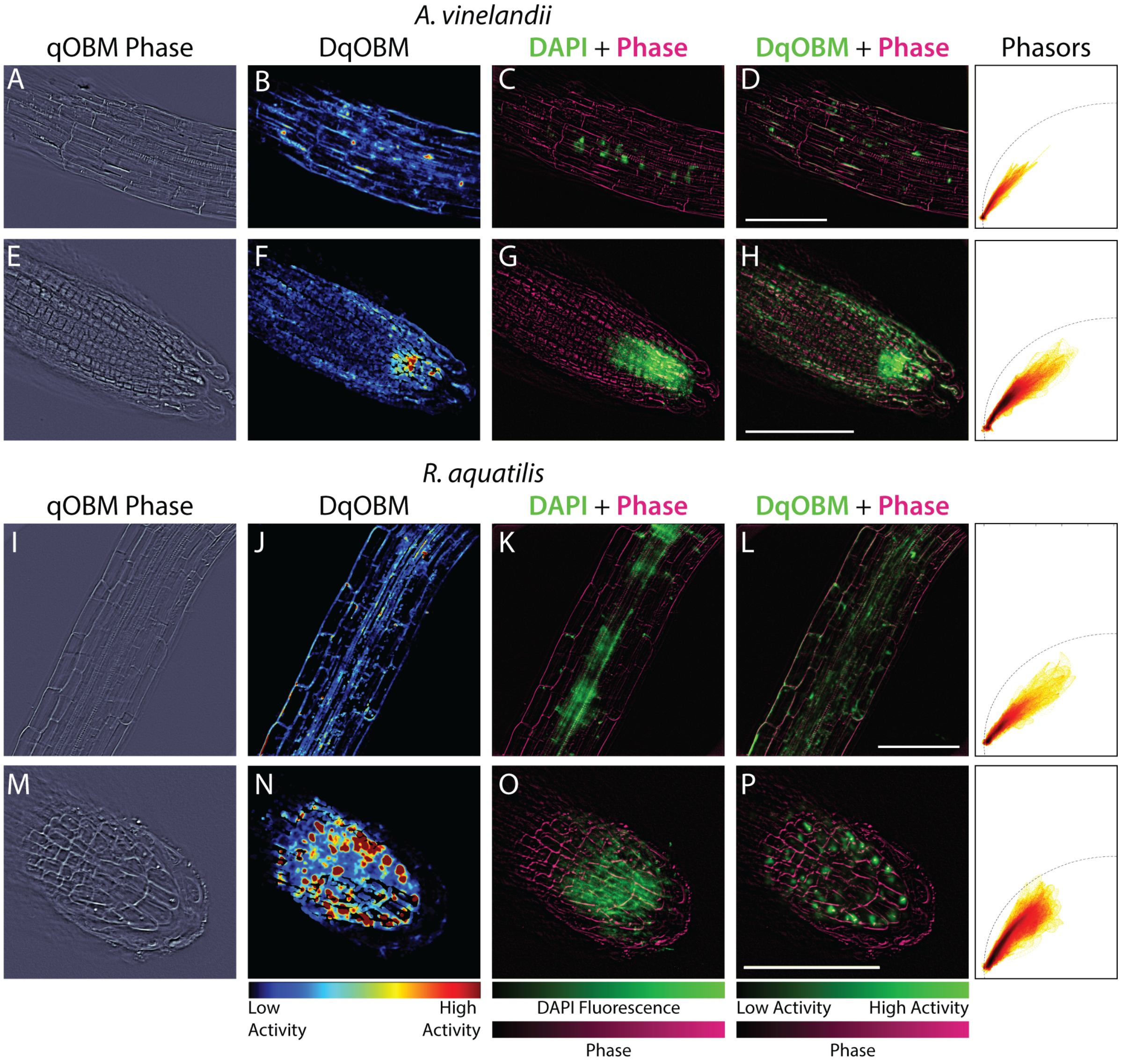

Figure S6. DAPI-labeled bacteria in plants. A-H show *A. vinelandii* inoculated plants. I-P show *R. aquatilis* inoculaated plants. A,E,I,&M show qOBM phase images, B,F,J,&N show DqOBM images, C,G,K,&O show DAPI fluorescence images (green) overlaid on qOBM phase images (magenta), and D,H,L,&P show DqOBM images (green) overlaid on qOBM phase images (magenta). We see a great alignment between the DAPI Fluorescence and DqOBM images. One major difference comes from the axial slicing performed in the imaging. DqOBM has a much higher axial slice sensitivity than DAPI. Thus, the DAPI fluorescence is not able to localize as well to a single phase image

Supplemental Section 5: Reported ANOVA Statistics

Below are the full row and column effects of the 2-way ANOVA analysis calculated from the data in Fig. 5.

ANOVA Analysis Variable	*A. vinelandii*	*R. aquatilis*	*S. meliloti*
Age Effect	$1.39 * 10^{-88}$	$8.94 * 10^{-53}$	$2.52 * 10^{-67}$
Nutrient Effect	$3.47 * 10^{-68}$	$1.67 * 10^{-77}$	$8.48 * 10^{-78}$
Interaction	$3.05 * 10^{-58}$	$2.00 * 10^{-44}$	$7.62 * 10^{-45}$

Table S1. ANOVA results of the analysis of the effects of the culture age, plated nutrients, and variable interaction. All show high significance.
